# Incomplete Spinal Cord Injury Reverses the Level-Dependence of Spinal Cord Injury Immune Deficiency Syndrome

**DOI:** 10.3390/ijms20153762

**Published:** 2019-08-01

**Authors:** James Hong, Alex Chang, Yang Liu, Jian Wang, Michael G. Fehlings

**Affiliations:** 1 Institute for Medical Science, University of Toronto, Toronto, ON M5S 1A8, Canada; 2 Krembil Research Institute, Toronto Western Hospital, Toronto, ON M5T 0S8, Canada; 3 Division of Neurosurgery, Faculty of Medicine, University of Toronto, Toronto, ON M5S 1A8, Canada

**Keywords:** spinal cord injury, cervical, thoracic, spleen, spinal cord injury-induced immune deficiency syndrome

## Abstract

Spinal cord injury (SCI) is associated with an increased susceptibility to infections, such as pneumonia, which is the leading cause of death in these patients. This phenomenon is referred to as SCI immune deficiency syndrome (SCI-IDS), and has been shown to be more prevalent after high-level transection in preclinical SCI models. Despite the high prevalence of contusion SCIs, the effects of this etiology have not been studied in the context of SCI-IDS. Compared to transection SCIs, which involve a complete loss of supraspinal input and lead to the disinhibition of spinally-generated activity, contusion SCIs may cause significant local deafferentation, but only a partial disruption of sympathetic tone below the level of injury. In this work, we investigate the effects of thoracic (T6-7) and cervical (C6-7) moderate–severe contusion SCIs on the spleen by characterizing splenic norepinephrine (NE) and cortisol (CORT), caspase-3, and multiple inflammation markers at 3- and 7-days post-SCI. In contrary to the literature, we observe an increase in splenic NE and CORT that correspond to an increase in caspase-3 after thoracic SCI relative to cervical SCI. Further, we found differences in expression of leptin, eotaxin, IP-10, and IL-18 that implicate alterations in splenocyte recruitment and function. These results suggest that incomplete SCI drastically alters the level-dependence of SCI-IDS.

## 1. Introduction

Traumatic spinal cord injury (SCI) is a devastating injury that has been shown to disrupt distal organs through maladaptive coordination of the hypothalamus–pituitary axis and the sympathetic nervous system **figure**. Recently, the phenomenon known as SCI-induced immunodeficiency syndrome (SCI-IDS) has been broadly implicated [1,2,3,4,5,6,7]. SCI-IDS is thought to be the cause of the increased susceptibility of SCI patients to pneumonia. The mechanisms by which the syndrome occurs is thought to be level-dependent, where high-level transection lesions (>thoracic level 3) cause a complete loss of supraspinal sympathetic flow to the spinal preganglionic neurons (SPNs) that innervate the adrenal gland and spleen. This loss of sympathetic flow ultimately results in several pathologies including splenic cortisol (CORT) and norepinephrine (NE) surge, severe leukopenia, splenic atrophy, and splenocyte apoptosis. These pathologies further coincide with the depression of circulating NE (adrenal insufficiency), elevation of circulating CORT, and an elevation of splenic NE and CORT 3-to-7 days post-injury [8,9,10]. A recent study characterized the pathways regulating SCI-IDS and found that while the deafferentation of adrenal glands accounts for the changes in NE and CORT (as it is the major producer), direct innervations did not account entirely for organ atrophy, as lymph nodes above the level of transection continue to experience cell loss due to circulating factors [5]. Using adrenalectomy and adrenotransplantation, the study demonstrated that organ atrophy after SCI is mediated through the spleen, and no level-dependence was shown for this phenomenon. At the spleen, the use of beta-2-adrenergic and glucocorticoid receptor antagonists have also been shown to rescue spleen atrophy [9], thus demonstrating that NE and CORT released via the maladapted sympathetic–neuroendocrine adrenal reflex plays a direct role in reducing splenocyte homing and apoptosis.

However, to date, SCI-IDS has been characterized exclusively in transection models of SCI. Full transections and hemisections are exceedingly rare in the clinic, as most patients suffer from incomplete lesions [11,12,13]. Outside of an increased susceptibility to pneumonia and adrenal insufficiency (e.g., requiring catecholamine administration), there exist no other robust parallel phenotypes (e.g., organ atrophy) between animal high-level transection models and cervical/high-thoracic patient populations [5]. Previously, we have reported no signs of splenic atrophy despite an overall reduction in circulating cytokine/chemokines after an incomplete cervical SCI [14]. On the contrary, at 14-days post-injury, we saw a significant increase in the spleen–body mass ratio relative to thoracic SCI. In the present study, we aimed to characterize splenic cytokine/chemokine, NE and CORT levels, and their impact on splenocyte apoptosis after incomplete cervical and thoracic SCI. In contrast to past literature, we demonstrate that incomplete lesions to the lower spine (T6–7; tSCI) result in profound splenocyte apoptosis, and elevations of both splenic NE and CORT relative to a cervical (C6–7; cSCI) lesion. These data suggest that the maintenance of partial supraspinal inputs in incomplete spinal cord lesions may drastically alter the level-dependency of SCI-IDS.

## 2. Results

### 2.1. Lesion Level-Dependent SCI Effects on Splenic Norepinephrine and Cortisol Levels

**Figure 1:**
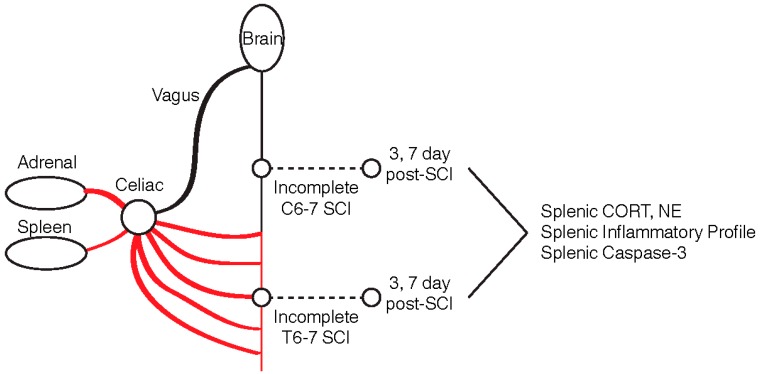
Experimental design. Two moderate-severe spinal cord injury (SCIs) were induced at cervical (C6–7) and thoracic (T6–7) levels and splenic cortisol (CORT), norepinephrine (NE), and inflammatory profile were assessed 3- and 7-days post-SCI.

**Figure 2:**
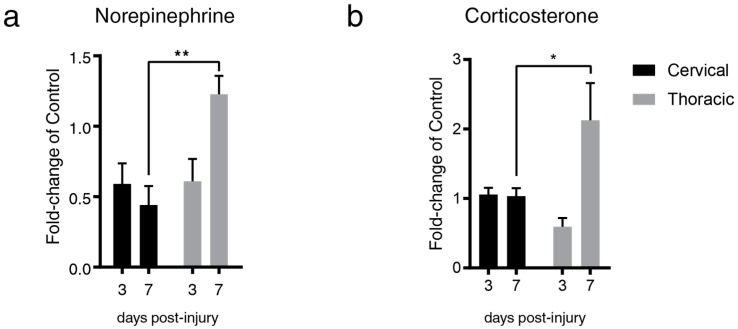
SCI results in changes to splenic NE and CORT in a level-dependent and time-dependent manner. (**a**) SCI at/below (T6–7) but not above (C6–7) the adrenal gland innervation results in marked elevation of splenic NE at 7-days post-injury (two-way ANOVA, ** *p* = 0.0025, t = 3.970, DF = 15, *n* = 5/group, 95% CI of diff. −1.28 to −0.29, Sidak’s multiple comparison test). (**b**) tSCI resulted in an elevation of splenic CORT 7-days post-injury relative to cSCI (two-way ANOVA, * *p* = 0.0301, t = 2.720, DF = 16, *n* = 5/group, 95% CI of diff. −2.08 to −0.10, Sidak’s multiple comparison test). Data shown as mean fold-change relative to time-matched control ± SEM (*n* = 3/control group).

We determined the kinetics of NE and CORT 3- and 7-days after a contusion-compression spinal cord injury. SCI was induced in rats at the C6–7 or T6–7 level with a modified aneurysm clip according to the experimental layout shown in Figure 1; time-matched laminectomized animals served as surgical controls. Based on the current literature, we expected splenic NE and CORT to be elevated after cSCI relative to tSCI, due to a release of NE from the adrenal medulla. On the contrary, we found reduced splenic NE at both levels 3-days post-injury (0.59-fold and 0.61-fold change relative to control for cSCI and tSCI, respectively), and significantly increased NE levels 7-days after tSCI relative to cSCI (0.44-fold and 1.24-fold change relative to control for cSCI and tSCI, respectively; Figure 2a). Similarly, while CORT was elevated after cSCI 3-days post-injury relative to control, it did not reach significance (1.05-fold and 0.59-fold change relative to control for cSCI and tSCI, respectively; *p* = 0.46). However, 7-days post-injury, splenic CORT was found to be significantly elevated in tSCI relative to cSCI (1.03-fold and 2.12-fold change relative to control for cSCI and tSCI, respectively; Figure 2b).

### 2.2. Lesion Level-Dependent SCI Effects on Splenocyte Apoptosis

**Figure 3:**
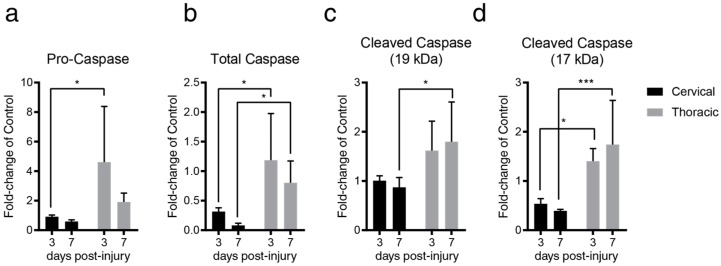
SCI results in changes to splenic pro-caspase-3, caspase-3, and cleaved caspase-3 in a level-dependent and time-dependent manner. (**a**) tSCI displayed a marked upregulation of splenic pro-caspase-3, 3-days post-injury relative to cSCI (two-way ANOVA, * *p* = 0.0156, t = 3.038, DF = 16, *n* = 5/group, 95% CI of diff. −6.69 to −0.69. Sidak’s multiple comparison test). (**b**) tSCI resulted in a marked elevation in splenic caspase-3 3-days post-injury relative to cSCI (two-way ANOVA, * *p* = 0.0137, t = 3.099, DF = 16, *n* = 5/group, 95% CI of diff. −1.56 to −0.17, Sidak’s multiple comparison test). Similarly, this difference persisted in a similar manner at 7-days post-injury (two-way ANOVA, * *p* = 0.0404, t = 2.574, DF = 16, *n* = 5/group, 95% CI of diff. −1.14 to −0.03, Sidak’s multiple comparison test). (**c**) tSCI, but not cSCI, resulted in elevated splenic cleaved caspase-3 (19-kDa form; two-way ANOVA, * *p* = 0.0378, t = 2.607, DF = 16, *n* = 5/group, 95% CI of diff. −1.80 to −0.04, Sidak’s multiple comparison test). (**d**) tSCI resulted in the elevation of splenic cleaved caspase-3 (17-kDa form), 3-days post-injury relative to cSCI (two-way ANOVA, * *p* = 0.0231, t = 2.848, DF = 16, *n* = 5/group, 95% CI of diff. −1.62 to −0.11, Sidak’s multiple comparison test). Similarly, this difference persisted in a similar manner at 7-days post-injury (two-way ANOVA, *** *p* = 0.0009, t = 4.417, DF = 16, *n* = 5/group, 95% CI of diff. −2.1 to −0.59, Sidak’s multiple comparison test). Data shown as mean fold-change relative to time-matched control ± SEM (*n* = 3/control group).

Next, we evaluated whether the surge in splenic NE and CORT 7-days post-tSCI resulted in splenocyte apoptosis. As we previously reported, we observed no significant change in the spleen–body mass ratio after cSCI and tSCI [14]. However, based on the profile of NE and CORT in tSCI, we anticipated an elevation in cleaved caspase-3 7-days post-tSCI. In line with this, we found a profound elevation of pro-caspase (0.91-fold and 4.6-fold change relative to control for cSCI and tSCI, respectively), caspase (0.31-fold and 1.18-fold change relative to control for cSCI and tSCI, respectively), and 17-kDa cleaved caspase-3 3-days post-tSCI relative to cSCI (0.54-fold and 1.4-fold change relative to control for cSCI and tSCI, respectively; Figure 3a,b,d). Further, we also found an elevation of caspase (0.08-fold and 1.18-fold change relative to control for cSCI and tSCI, respectively), as well as the 19-kDa (0.87-fold and 1.79-fold fold change relative to control for cSCI and tSCI, respectively), and 17-kDa cleaved caspase (0.39-fold and 1.74-fold change relative to control for cSCI and tSCI, respectively), 7-days post-tSCI relative to cSCI (Figure 3b–d).

### 2.3. SCI induces Level-Dependent Splenic Cytokine/Chemokine Changes

**Figure 4:**
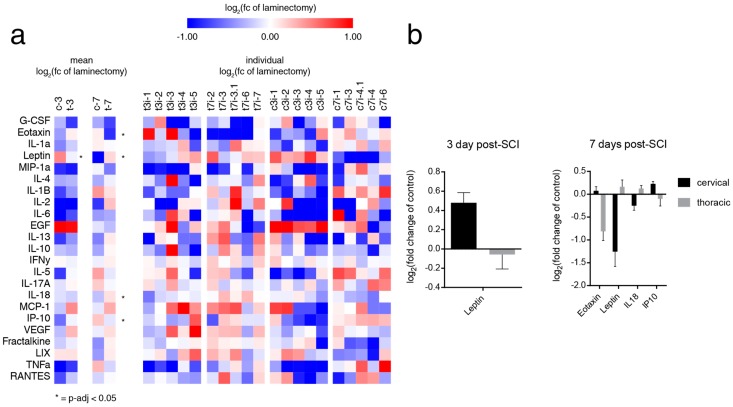
SCI induces changes in the splenic expression of cytokine/chemokines in a level-dependent and time-dependent manner. (**a**) Heatmap demonstrating both the individual and the mean log_2_ fold change of control after cSCI and tSCI. At 3-days post-injury, tSCI had relatively lower levels of leptin compared to cSCI (Kruskal–Wallis, * *p* = 0.028, t = 4.811, DF = 1, *n* = 5/group, all pairwise comparisons). At 7-days post-injury, tSCI had relatively lower levels of eotaxin (Kruskal–Wallis, * *p* = 0.028, t = 4.840, DF = 1, *n* = 5/group, all pairwise comparisons) and IP10 (Kruskal–Wallis, * *p* = 0.047, t = 3.938, DF = 1, *n* = 5/group, all pairwise comparisons) compared to cSCI. Contrastingly, tSCI had relatively elevated levels of leptin (Kruskal–Wallis, * *p* = 0.009, t = 6.818, DF = 1, *n* = 5/group, all pairwise comparisons) and IL18 (Kruskal–Wallis, * *p* = 0.016, t = 5.771, DF = 1, *n* = 5/group, all pairwise comparisons). (**b**) Bar graphs showing only the cytokines/chemokines that were significantly different between the levels at 3- and 7-days post-injury. Data shown as mean fold change of time-matched control ± SEM (*n* = 3/control group).

Finally, we examined the cytokine and chemokine profile of the spleen after cSCI and tSCI (Figure 4a). In our previous work, we showed a profound reduction in circulating interferon γ-induced protein 10 (IP10) 3- and 7-days following tSCI relative to cSCI, which is a key molecule in regulating T-cell generation and trafficking. On the contrary, the current literature suggests that high-level SCI perturbs lymphocyte—particularly B-cell—homing to the spleen. As we observed stark reversals in both NE/CORT and caspase, we anticipated that tSCI would result in a depletion of leukocyte/lymphocyte chemotaxins in the spleen. Indeed, at 3-days post-tSCI, both IP10 and eotaxin (also known as CCL2, a potent leukocyte chemoattractant) were significantly depressed relative to cSCI, which displayed contrary expression (Figure 4b). Intriguingly, tSCI also displayed significantly increased leptin (0.48-log_2_ fold and −0.05 log_2_ fold change of control for cSCI and tSCI, respectively)—a regulator of energy intake and expenditure known for suppressing splenic lymphocyte function—as well as interleukin 18 (IL18)—an inducer of interferon- γ (IFN-γ) in T and NK cells—7-days post-injury relative to cSCI (Figure 4b).

## 3. Discussion

### 3.1. Incomplete SCI Alters Level Dependence of Several Hallmarks of SCI-IDS through Localized Denervation and Partial Preservation of Sympathetic Tone

Our study is the first study to demonstrate that cervical and thoracic compression/contusion spinal cord lesions may differentially influence the development of SCI-IDS. While other studies have not examined both levels concurrently, there have been studies of low thoracic incomplete lesions that have reported similar results. For instance, Zha et al. found a profound elevation of NE following a chronic thoracic level 9 contusion, and functional deficiencies in the T-cell population without any signs of splenic atrophy [15]. Similarly, Norden et al. found that impaired T-cell antiviral immunity follows an acute T9 contusion alongside elevated CORT levels [16]. These studies demonstrate the complexities of sympathetic disruption, and how partial deafferentation of the adrenal and splenic innervations can result in a level dependency that is unlike that of transection SCI. We posit that in an incomplete injury, the sympathetic tone below the level of the injury may be reduced or altered, but not eliminated. It is this factor, in conjunction with the well-documented plasticity and rewiring, that may result in preserved splenic and adrenal innervation after cSCI that continues to respond to the injury stimulus [4]. However, at the lesion site, there is severe tissue loss that results in cystic cavitation as early as 3-days post-injury. This cavity undoubtedly disrupts the local circuitry and may cause—as seen in our data and the T9 contusion studies above—symptoms that mimic local deafferentation of the preganglionic SPNs that lead to the spleen and adrenal gland.

### 3.2. Elevations in Splenic Norepinephrine and Corticosterone Result in Splenocyte Apoptosis and a Reduction of Key Leukocyte and Lymphocyte Generation and Trafficking Molecules

There are several studies that document the impact of both NE and CORT on IP10, leptin, IL18, and eotaxin. For instance, CORT has been implicated in the inhibition of multiple leukocyte chemoattractants, including eotaxin, eotaxin-2, and monocyte chemotactic protein 4 [17]. While eotaxin serves as a potent chemoattractant and homing signal for leukocytes, IP10 is known to be critically involved in effector T-cell generation and trafficking [18]. IP10-deficient mice have been shown to have decreased proliferation and weaker IFN-γ secretion in response to immune challenge. On the other hand, leptin is a potent regulator of energy intake and expenditure, and has also been shown to suppress splenic T-cell function through the activation of corticotropin-releasing hormone [19,20,21]. Further, CORT has been shown to promote leptin secretion. Finally, IL18 has been shown to be induced by agonism of B_2_AR and by elevated serum CORT [22,23]. IL18 exerts its effect by stimulating IFNγ secretion from splenocytes; thus, it is likely that its elevation signals an attempt for splenic IFNγ production to induce various T-cell trafficking and generating molecules downstream (including IP10).

Despite adequate controls, a caveat that must be considered is that of anesthesia and clavamox, and its potential for differential synergistic/antagonistic effects after cSCI and tSCI. Although no documented effect on sympathetic tone has been shown with clavamox, it is well known that induction with isoflurane is accompanied by hypotension, indicating an attenuation of sympathetic tone [24]. The accompanying hypotension may synergize with the systemic hypotension following cSCI (and not tSCI), which may impact circulation and overall physiology to some extent. Despite this, there is no evidence to suggest that there are significant alterations to the splenic perfusion following isoflurane induction.

Taken together, our data shows that an incomplete tSCI, but not cSCI, displays an aberrant sympathetic–neuroendocrine coordination that closely mimics key hallmarks of SCI-IDS. This may suggest two possible scenarios that complement the current mechanisms proposed by the literature: (1) a partial loss of sympathetic tone below the site of injury is insufficient to instigate deafferentation of the SPNs; and (2) SPNs branching from the lesion site are lost or severely disrupted to the degree of deafferentation. Taken together, these mechanisms could explain how tSCI instigates SCI-IDS due to a loss of local circuitry, while cSCI remains unaffected due to the preservation of sympathetic tone below the site of injury.

## 4. Materials and Methods

All the animal experiments were approved by the animal care committee at the University Health Network (Toronto, Ontario, Canada) in compliance with the Canadian Council on Animal Care (Project ID Code: #2212, Date of Approval: 17 May 2017).

### 4.1. Clip-Compression SCI and Spleen Extraction

The same cohort of female adult Wistar rats (12 weeks old, 250–300 g, *n* = 5/group for injured, *n*= 3/group for laminectomy) were used, as previously described [14]. Prior to surgery, 0.05 mg/kg of buprenorphine and 5 mL of saline were administered subcutaneously. Then, 1–2% of isoflurane in a 1:1 mixture of O_2_ and N_2_O were used for anesthesia, and a laminectomy was performed at C6–7 or T6–7. Following the laminectomy, a moderate–severe injury was induced for 1 min at the cervical or thoracic level, as described previously [14]. Until the endpoint (3- and 7-days post-SCI), the animals were given subcutaneous buprenorphine (0.05 mg/kg, bid), oral amoxicillin trihydrate/clavulanate potassium (Apotex Pharmaceuticals, Toronto, ON, Canada), and subcutaneous saline injections (5 mL on each side of the animal). Animals were housed individually in cages at 27 °C, and their bladders were manually expressed thrice daily. Prior to sacrifice and perfusion, spleens were collected from anesthetized rats, and their weights were recorded and normalized to their body mass. Then, spleens were flash frozen in liquid nitrogen and stored in −80°C.

### 4.2. Protein Extraction from the Spleen

Frozen spleens were crushed with a mortar and pestle over dry ice with constant pouring of liquid nitrogen. The pellet was collected by swirling the liquid nitrogen and spooning the consolidated pellet into a 1.5-mL protein Lo-Bind tube (Eppendorf, Germany). Then, the pellets were mixed with 1xRIPA buffer (Thermo Fisher Scientific, MA, USA) at a ratio of 4 mL per g of tissue followed by two rounds of 10-s sonication to homogenize the tissue. Then, the samples were centrifuged at 15,000 rpm for 15-min and the protein supernatant was taken. Then, the protein was quantified using the microBCA kit (Thermo Fisher Scientific, MA, USA), where the 562-nm reading was used to map the sample absorbance against known concentrations of bovine serum albumin.

### 4.3. Quantitative ELISA for Norepinephrine and Corticosterone

Splenic NE and CORT levels were measured using quantitative ELISA kits. First, 50 ug of protein was used to quantify samples using the NE ELISA kit (KA1891, Abnova, Taipei, Taiwan) and 10 ug of protein was used to quantify samples using the CORT ELISA kit (ab108665, Abcam, Cambridge, United Kingdom). For both kits, 450 nm was used to quantify the absorbance values of the samples (620 nm was subtracted as background for the NE kit, while 650 nm was subtracted as a background of the CORT kit). The standard curve was fitted to a four-parameter logistic regression on GraphPad Prism, as suggested by the manual, and the absorbance values of the samples were mapped to the concentrations based on known concentrations of NE and CORT.

### 4.4. Western Blot

Thirty ug of protein was used for Western blotting. The proteins were first mixed in with 2× lamelli loading buffer (Biorad, Hercules, CA, USA) with 2-mercaptoethanol and boiled at 95 °C for 5 min. Then, the samples were loaded into 4–20% gradient gels (Biorad, Hercules, CA, USA) and run at 250-V constant voltage until the dye front reached the edge of the gel cast. Then, the proteins were transferred at 100 V for 1 h unto 0.2-µm nitrocellulose membrane (Biorad, Hercules, CA, USA). A reversible Ponceau-S stain was performed and captured on the BioRad ChemiDoc scanner to be used for total protein normalization. Then, the Ponceau-S stain was removed by gentle washing with 1× TBST, and the membrane was blocked in 4% skim milk in 1×TBST for 1-hour at room temperature. Finally, the caspase-3 antibody (9662, 1:1000, Cell Signaling Technology, Danvers, MA, USA) and cleaved caspase-3 Asp175 antibody (9661, 1:1000, Cell Signaling Technology, Danvers, MA, USA) were used for overnight 4 °C incubation. The next day, the membranes were incubated in anti-rabbit HRP secondary (1:1000, Sigma-Aldrich, St. Louis, Missouri, USA) and auto-exposed on the ChemiDoc scanner (Biorad, Hercules, CA, USA).

### 4.5. High-Throughput Luminex Array for Cytokines and Chemokines

A total of 250 µg of protein were sent to Eve Technologies (https://www.evetechnologies.com) for high-throughput luminex profiling using their rat Discovery Assays™ for cytokine/chemokines (RD27). All the proteins that contained interpolated/extrapolated/out-of-range values were removed from the study. The concentration of these proteins was calculated using a standard curve and expressed in pg/mL.

### 4.6. Statistical Analysis

Heatmapping was performed using the Morpheus software package from the Broad Institute (Cambridge, MA, USA, https://software.broadinstitute.org/morpheus/), and graphing was done using GraphPad Prism (La Jolla, California, USA). An assessment of normality was performed for each group using the Shapiro–Wilk test of SPSS (IBM, Armonk, New York, USA). All the comparisons between cSCI and tSCI were performed in SPSS using either the one-way or two-way ANOVA function with post hoc Sidak’s for multiple corrections (parametric, *p*-adjusted threshold = 0.05) or Kruskal-Wallis test with post hoc Dunn’s for multiple corrections (non-parametric, *p*-adjusted threshold = 0.05).

## Abbreviations

SCI	Spinal cord injury
SCI-IDS	Spinal cord injury-induced immunodeficiency syndrome
NE	Norepinephrine
CORT	Corticosterone
tSCI	Thoracic spinal cord injury
cSCI	Cervical spinal cord injury
SPN	Spinal preganglionic neurons
TBST	Tris-buffered saline with 0.1% Tween-20
GMCSF	Granulocyte-macrophage colony-stimulating factor
IL1α	Interleukin 1 alpha
IL1β	Interleukin 1 beta
IL2	Interleukin-2
IL17A	Interleukin-17A
IL18	Interleukin-18
MCP1	Monocyte chemoattractant protein-1
IP10	Interferon gamma-induced protein 10
LIX	Lipopolysaccharide-induced CXC chemokine
MIP2	macrophage inflammatory protein 2
RANTES	regulated on activation, normal T cell expressed and secreted
GCSF	Granulocyte - colony stimulating factor
IL4	Interleukin-4
IL10	Interleukin-10
IL12	Interleukin-12
IL5	Interleukin-5
